# Towards a Clustering Guided Hierarchical Framework for Sensor-Based Activity Recognition

**DOI:** 10.3390/s21216962

**Published:** 2021-10-20

**Authors:** Aiguo Wang, Shenghui Zhao, Huan-Chao Keh, Guilin Chen, Diptendu Sinha Roy

**Affiliations:** 1 School of Electronic Information Engineering, Foshan University, Foshan 528225, China; wangaiguo2546@163.com; 2 School of Computer and Information Engineering, Chuzhou University, Chuzhou 239000, China; zsh@chzu.edu.cn (S.Z.); glchen@chzu.edu.cn (G.C.); 3 Department of Computer Science and Information Engineering, Tamkang University, New Taipei City 25137, Taiwan; 4 National Institute of Technology, Shillong 793003, India; diptendu.sr@nitm.ac.in

**Keywords:** wearable computing, activity recognition, clustering guided

## Abstract

Human activity recognition plays a prominent role in numerous applications like smart homes, elderly healthcare and ambient intelligence. The complexity of human behavior leads to the difficulty of developing an accurate activity recognizer, especially in situations where different activities have similar sensor readings. Accordingly, how to measure the relationships among activities and construct an activity recognizer for better distinguishing the confusing activities remains critical. To this end, we in this study propose a clustering guided hierarchical framework to discriminate on-going human activities. Specifically, we first introduce a clustering-based activity confusion index and exploit it to automatically and quantitatively measure the confusion between activities in a data-driven way instead of relying on the prior domain knowledge. Afterwards, we design a hierarchical activity recognition framework under the guidance of the confusion relationships to reduce the recognition errors between similar activities. Finally, the simulations on the benchmark datasets are evaluated and results show the superiority of the proposed model over its competitors. In addition, we experimentally evaluate the key components of the framework comprehensively, which indicates its flexibility and stability.

## 1. Introduction

Benefiting from the rapid development and fusion of sensing technology, pervasive computing, and artificial intelligence, researchers have designed and implemented a wealth of Internet of Things (IoTs) systems and applications, such as behavior analysis, sports and games, the elderly healthcare, chronic disease management, smart buildings, smart homes and ambient intelligence, etc. [1,2]. From the perspective of system architecture, these application services have a typical three-layer architecture of IoTs (sensing layer, network layer, and application layer) [3,4]. With the complex characteristics of human behavior, it is not very easy to build an accurate activity recognizer for practical applications [5]. For example, there exist intra-subject and inter-subject variations of how people perform an activity, which is difficult to the generalization ability of activity recognizers [6]. Besides performing activities sequentially, an individual can conduct concurrent and interleaved activities, and there are activities involved in a specific application having similar sensor signals, which confuses an activity recognizer to a certain extent [7,8]. Therefore, automatically and accurately recognizing human activities is still a challenging issue [9,10].

In the literature, many activity recognition models had been proposed. They were categorized into three types based on the technology, including vision-based, environment interaction-based, and wearable-based methods [11,12,13]. Vision-based mechanisms exploit the computer vision techniques to analyze and infer the human activities in camera captured content. The recent years have witnessed enormous growth in their applications such as video surveillance, games, and security, while their performance is sensitive to background noise and illumination variation [14]. Moreover, their use often raises privacy issues and is further limited to the fixed places [11]. For the second type methods, they analyzed the on-going activities by recording the interaction between the person and the surroundings via the sensing units placed on household objects. Obviously, they are usually limited to indoor scenarios [15]. Different from the above two types of methods, wearable-based methods largely benefit from the communication capabilities of wearable devices. Typically, they first collected the sensor data when one performed activities and trained an activity recognizer, which was used for making predictions [16]. That is to say, a person could wear multiple homogeneous or heterogeneous sensing devices on the body [17], which was more suitable for both scenarios.

In terms of the activity recognition models, there were many models from the generative and discriminative models to unsupervised learning, ensemble learning, and deep learning models [18,19,20]. Although these models advance the study of human activities, most of the studies recognized the predefined activities in a one-step, which we called the flat model for illustration purposes. However, for the activity recognition supported applications, different activities (even if they have totally different semantics) could have quite similar sensor signals, which reduced the ability of activity recognizers in distinguishing confusing activities and inevitably led to degraded accuracy [7,8]. One feasible solution is to develop a hierarchical activity recognition model in order to derive a superior decision between similar activities towards performance improvement, where there are two key factors that determine its practical use and performance. First, determining the relationships among activities is a prerequisite for constructing a hierarchical activity recognizer. Different from the simple and easy case where we can use prior knowledge to group activities, it is a challenging task for new and under-explored cases with few or even no domain expert knowledge about the activities available for use. Accordingly, how to quantitatively measure the confusion among activities largely influences its generalization ability across different scenarios. Second, how to effectively utilize the relationship to build an activity recognizer affected its overall performance in analyzing confusing activities. This requires us to develop a model to better distinguish different activities and to maximize the decision distance between confusing activities. To handle the key issues, we herein propose a clustering guided hierarchical activity recognition framework that consists of two critical components. Specifically, we first introduce a clustering-based activity confusion index and use it to quantitatively measure the confusion among activities in a data-driven way. Afterwards, we develop a hierarchical activity recognition model under the guidance of the relationships to reduce the recognition errors between similar activities. The contributions of the proposed study are listed. (1) Instead of depending on prior knowledge, a data-driven approach, which quantitatively measures the confusion among activities has been proposed. This potentially facilitates the development of the hierarchical activity recognition model and its extension to new and under-explored situations that have limited prior domain knowledge. (2) We formalize the problem and accordingly propose a hierarchical human activity recognition framework guided by the proposed activity confusion index, which has the capability of connecting any two activities and returning a superior decision between activities having similar sensor signals. This is expected to reduce confusion errors. (3) We also experimentally investigate the power of tri-axial accelerometers and gyroscopes and study the parameter sensitivity of the components of the framework, which indicates the power of fusing distinct sensors and the flexibility and stability of the proposed model.

The structure of this paper is shown as follows. Section 2 discusses the related work on activity recognition models. The proposed clustering guided hierarchical framework is given in Section 3. Section 4 and Section 5 analyze the experimental settings and the performance results. The conclusion is given in Section 6.

## 2. Related Work

Different from image processing and text analysis, the development of activity recognition includes many challenges such as modeling, evaluation, and human behavior, which is characterized by inherent complexity. To get enhanced performance and generalization ability across different scenarios, researchers have proposed many activity recognition models, which can be categorized into two categories: knowledge-driven and data-driven approaches [6,21,22].

Knowledge-driven approaches utilized domain knowledge for activity specification and further used it for sensor modeling, context fusion, activity modeling and recognition [23]. Commonly used modeling tools include, but are not limited to, ontology modeling, evidential theory, and logical modeling [24]. For example, an activity recognition model based on the ontologies and reasoning is used to infer on-going activities [21]. They implemented the proposed approach in a software system and evaluated it as a smart home research laboratory and achieved an overall accuracy of 94.44%. Although the knowledge-driven approaches have advantages of high efficiency, robustness to noise, and easy interpretation, they usually need domain expert knowledge for activity and context modeling, which is not a trivial task, especially for a new and underexplored domain.

Data-driven approaches, also called data-centric approaches, aim to learn an activity recognition model from the collected sensor data [25]. Study [16] used *k* nearest neighbors and naïve Bayes to train sensor-based activity recognizers. Another study [26] utilized the random forest algorithm to recognize activities such as *jump*, *run*, *static*, *walking*, *go-upstairs*, and *go-downstairs*. There are also researches that use deep learning models to jointly optimize the feature learning and classifier training [27,28,29]. Study [30] proposed a deep convolutional neural network (CNN) based activity recognizer to predict the activities based on the mobile phone data. Ordóñez and Roggen introduced a convolutional long short-term memory (LSTM) model, and they experimentally evaluated its power, demonstrating its superiority over its competitors [17].

Besides, some studies combine knowledge-driven and data-driven approaches. Azkune et al. proposed a model that uses the data-driven approach to evolving knowledge-driven approach aiming to obtain knowledge-driven activity recognition models [31]. Sukor et al. presented a hybrid activity recognition model that first used knowledge-driven reasoning to get an initial activity model and then optimized it using a data-driven approach. They experimentally evaluated the proposed model, which showed significantly higher recognition rates [32].

To better handle the activities that have similar sensor signals, some studies present the hierarchical activity recognition models. Different from the flat models that classify the activities of interest in a single step, hierarchical models usually adopt a coarse-to-fine scheme to infer the activity label via a multi-stage decision, where they transform the original problem into a series of classification problems [33]. Study [25] developed a hierarchical activity recognition model that recognized human gestures and predicted the final activity. Liu et al. presented an activity recognition model that recognized the actions and then utilized the temporal patterns of the actions to recognize activities [34]. From the perspective of the prediction process, we call such a scheme the *bottom-up* approach. Instead, some studies work in a *top-down* scheme, where it first predicts an abstract activity and then infers the specific label of the activity. Study [35] proposed a two-stage model that classified activities into dynamic, static, or transition states and then inferred the specific activity within the activity group. Cho and Yoon proposed an activity recognition model based on two-stage 1D CNN to first predict the test sample within an abstract activity group and then make predictions within the group [8]. Wang et al. used prior domain knowledge of the activities to manually organize them into a tree structure and proposed a tree-based activity recognizer [7]. Although top-down methods make no assumption about the temporal relationship and obtain enhanced performance, most of the studies rely on prior knowledge about the activities to group activities and to build a hierarchical activity recognizer [7,8,34,36]. This hinders them when handling new and complex scenarios of which there is little expert knowledge. Moreover, wrong grouping can result in degraded accuracy. In addition, one major limitation of the tree-based models is that they probably induce the accumulation of errors in the hierarchical predictions. Therefore, the above issues motivate us to explore the data-driven way to automatically and quantitatively measure the relationships among activities and then build an activity recognizer for reducing the accumulation of errors. Accordingly, we present a clustering guided hierarchical activity recognition model. Compared with previously work, our proposed data-driven model utilizes the clustering theory to identify confusing activities and quantifies the relationships among activities with the two proposed confusing indices (i.e., cluster confusion index and activity confusion index). Different from the tree-based model, our proposed model allows the flexible connections between any two activities, which helps return a good decision boundary between similar activities. Particularly, the proposed method is a general framework that can take as its building blocks various clustering and classification models and even deep learning models.

## 3. Clustering Guided Activity Recognition Framework

The characteristics of the complex human behavior lead to the difficulty of designing an activity recognizer, specifically in handling cases where activities with different semantics have similar sensor readings. As a result, this degrades the discriminant capability of an activity recognizer. For example, we experimentally observe that the sensor readings between dynamic activities are more similar than the ones between static activity and dynamic activity. Hence, training a flat activity recognizer to classify the three activities pays much attention to the decision boundary between static activity and dynamic activities and fails to determine a good decision between the two dynamic activities.

**Figure 1:**
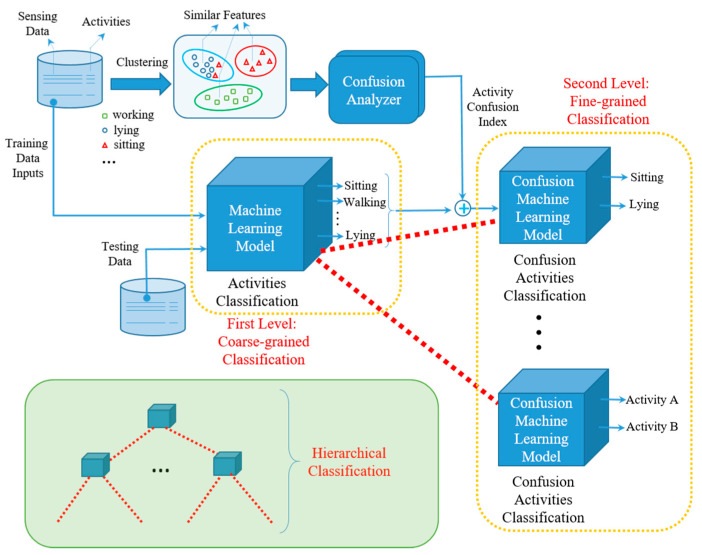
The Flowchart of the proposed model.

One feasible solution is to construct a hierarchical activity recognizer, where we infer the activity labels in a multi-stage way. Accordingly, the key is how to determine the relationships among activities and use them to direct the design of an activity recognition model, especially for the case with little or no prior knowledge. Figure 1 depicts the proposed clustering guided hierarchical activity recognition model, which consists of two-level classification. The training data set contains two parts: the sensing data and the label of activity. Initially, the sensing data will be the input of the designed model, which is the first level of coarse-grained classification. Since the sensing data of some activities, such as sitting and lying, are similar, they might fall in the same cluster. This occurs because their features represented by the sensing data are similar. To further distinguish them in a way of fine-grained feature extraction, a Confusion Analyzer is designed to assign each activity with an activity confusion index, which represents the similarity between activities. The output of the first level coarse-grained classification will further play the role of input data of the second level fine-grained classification model, aiming to further distinguish similar activities. According to the assigned activity confusion index, the output of the first level classification model can be the final decision without feeding into the lower-level classification, if it does not fall in the same group of other activities. Otherwise, the output of the first level coarse-grained classification model will be further processed by applying the fine-grained classification. Herein, the component of Confusion Analyzer aims to assign each activity with an activity confusion index according to the clustering results. The coarse-grained classification and fine-grained classification could use the same machine learning model to design their recognizers. For a specific application, we train a coarse-grained classification model and selectively train a fine-grained classification model for each activity. That is, if there exist confusing activities for one activity, we train a fine-grained model associated with it. Accordingly, the number of classes to be predicted for coarse-grained classification and fine-grained classification is different, and the coarse-grained model usually has higher storage and computation complexity. Although the example given in Figure 1 only depicts the two-level activity recognition. However, the general model of hierarchical classification, as shown in the block of the left bottom of Figure 1, can have multi-level classification models where the number of levels is larger than two.

The framework contains two core components: determining the confusion among activities, which is implemented by the Confusion Analyzer, and constructing a hierarchical model. The idea is to build the hierarchy of the predefined activities under the guidance of the relationships among activities, which is determined by the Confusion Analyzer. Specifically, we first measure the activity relationships without the reliance on prior knowledge by introducing a clustering-guided confusion index. Guided by the relationships, we then design and train a hierarchical activity recognition model. Finally, we apply it to recognize on-going activities associated with the sensor signals. In the next subsections, we detail the former two components. For illustration purposes, *D* denotes the training set, and *L* states the predefined activity labels. *D* = (*X*, *Y*) is a labeled dataset, where *X* is the feature matrix and *Y* is the corresponding label of each sample of *X*. *tx* is a test sample, and |*L*| indicates the number of predefined activities.

### 3.1. Measuring the Confusion among Activities

Considering that similar activities tend to generate similar sensor signals from the view of data distributions, we utilize the idea of clustering techniques to partition the data points of the predefined activities into multiple groups and analyze the group purities to quantify the confusion among activities. We herein introduce two definitions before illustrating the procedure.

**Definition** **1.** *(Cluster Confusion Index). Given a cluster C consisting of a subset of samples from D, the class of C is set as the label L_i_ (1 ≤ i ≤ |L|) that has the maximum data points in C. The number of samples with label L_j_ (1 ≤ j ≤ |L|, i ≠ j) is defined as the cluster confusion index between L_j_ and L_i_ and is referred to as conf_c_(L_j_ → L_i_).*

(1) $$L_{i}=\underset{L_{k}\in L}{\max}\{\sum_{x\in C}I(y_{x}=L_{k})\},$$

*where I (a = b) is an indicator function that returns 1 only if a equals b, x is a sample of C, and y_x_ is the class label of x. The conf_c_(L_j_ → L_i_) indicates how many samples of L_j_ in C are labeled as L_i_ and denotes the confusion between L_j_ and L_i_ in C. We can then measure the overall confusion index between L_j_ and L_i_ in D.*

**Definition** **2.** *(Activity Confusion Index). Given the k clusters obtained by manual assignment or returned by a clustering algorithm, the activity confusion index conf(L_j_ → L_i_) between L_j_ and L_i_ is defined as the sum of cluster confusion index of the k clusters, as given in Equation (2).*

(2) $$conf(L_{j}\to L_{i})=\sum_{c=1}^{k}conf_{c}(L_{j}\to L_{i})$$

According to Definitions 1 and 2, we can apply a data-driven or knowledge-driven approach on *D* to getting *k* clusters and further obtaining the confusion index *conf*(*L_j_* → *L_i_*) between *L_j_* and *L_i_*. Due to the advantage of the data-driven approach over the knowledge-driven approach, we exploit the power of clustering techniques in partitioning *D*.

Next, when considering each pair of the predefined activities, we can organize the activity confusion index into a confusion matrix *CM*, also called the relationship matrix. Let *CM* denote a square matrix, and the number of rows equals *|L|*. The entry *CM_ji_* of the *j*-th row and *i*-th column is the samples from the *j*-th activity that is categorized as the *i*-th activity. A large value of *CM_ij_* leads to more confusion between the activities.

After quantifying the relationships among activities, we then detail how to use them to direct the construction of an activity recognition model in better discriminating confusing activities.

### 3.2. Hierarchical Activity Recognition Model

The above designed activity confusion matrix indicates that if the predicted label of a test sample *tx* is *L_i_*, its true label probably comes from *L_j_*. We herein use a confusion threshold *θ* to decide whether *L_j_* is a potential confusing activity of *L_i_*. Specifically, for the *i*-th column, if the ratio *η*(*L_j_*, *L_i_*) of *CM_ji_* to the sum of the *i*-th column of *CM* exceeds *θ*, *L_j_* is taken as a confusing activity of *L_i_* and a mechanism is expected to distinguish *L_j_* and *L_i_*.

(3) η(Lj,Li)=CMji∑j=1|L|CMji≥θ

Particularly, for each activity *L_A_* ∈ *L*, we can find a subset of activities *S*(*L_A_*) that are the confusing activities of *L_A_*. That is, it is necessary to further distinguish between *S*(*L_A_*) and *L_A_* if the predicted activity label is *L_A_* in the first step. Accordingly, we propose a hierarchical human activity recognition framework that works in the following scheme. In the training phase, initially, a top-level classification model *cls_all* are trained to differentiate among all the predefined activities, which takes essentially the same steps of the flat model. Afterwards, for each activity *L_A_* ∈ *L*, whose *S*(*L_A_*) is not empty, then a second-level classifier *cls_L_A_* is trained to classify *L_A_* and *S*(*L_A_*). Note, *S*(*L_A_*) is obtained from the activity confusion matrix in the previous step. In the prediction stage, given a test sample *tx*, we first classify it using *cls_all* and get the predicted result *L_A_*. If *S*(*L_A_*) is not empty, we then apply *cls_L_A_* on *tx* to return the inferred label; otherwise, report *L_A_*.

According to subsections A and B, the proposed framework is shown in Algorithm 1. The lines 1–2 show the steps of quantifying the confusion among activities, lines 3–5 denote the classification model training that mainly describes how to build a hierarchical activity recognizer with the guidance of the activity relationships, and lines 6–8 show the procedure of obtaining the predicted label of a test sample, which involves two-level classifications. Particularly, we present the roles of the two core components in Algorithm 1, where lines 1–2 illustrate the first component and lines 3–5 correspond to the second component, to state how to integrate them into the proposed framework.
**Algorithm 1:** Clustering Guided Hierarchical Activity Recognition Framework. **Input:** Training set *D* and labels *L* for activities, Threshold *θ*, test set *tx***Output:** The prediction of activity with label *L_A_* of *tx**// TRAINING STAGE*1. Divide *D* into groups *CLU*; // **Component #1**2. calculate the activity confusion matrix *CM* of *CLU* by applying (1) and (2);3. Construct *cls_all* to identify all activities; // **Component #2**4. **for** each activity *L_A_* of *L* **do**
   (4.1) *S*(*L_A_*) = { }; **//** initialization of *L_A_*5. **for** each activity *L_A_* of *L* **do**   (5.1) **for** each activity *L_B_* of *L* **do**      obtain *η*(*L_B_*, *L_A_*) by applying (3);       **if** *L_A_* != *L_B_* and *η*(*L_B_*, *L_A_*) ≥ *θ*
**do**        *S*(*L_A_*).add(*L_B_*);    (5.2) **if** not_empty(*S*(*L_A_*)) **do**        construct a *cls_L_A_* to distinguish *L_A_* and *S*(*L_A_*);*// PREDICTION STAGE*6. *L_A_ = cls_all* (*tx*); // infer the label of *tx* by applying the top-level classifier7. **if** not_empty(*S*(*L_A_*)) **do**    *L_A_ = cls_L_A_*(*tx*); // infer the label of *tx* by applying the second-level classifier8. **return** *L_A_*


## 4. Experimental Setup

### 4.1. Experimental Data

This section evaluates the comparative experiments on the UCI-HAR dataset, which consists of six human activities [37]. The smartphone, which has a 3-axis accelerometer and a gyroscope, works with a sampling rate of 50 Hz. For illustration purposes, X, Y, and Z refer to the three axes of the sensing unit, and ‘t’ (‘f’) denotes the time domain information. The features are associated with raw accelerometer signals tAcc-XYZ and gyroscope signals tGyto-XYZ. The frequency-domain features are obtained by applying a Fast Fourier Transform on tAcc-XYZ, and we get fBodyAcc-XYZ and fBodyAccJerk-XYZ. Similarly, for the gyroscope, we extract Jerk signals tBodyGyroJerk-XYZ, tBodyGyroJerkMag, and tBodyGyroMag from tGyto-XYZ. We then extract the frequency-domain features fBodyGyro-XYZ, fBodyGyroMag, and fBodyGyroJerkMag. In summary, 348 and 211 features are extracted from the accelerometer and gyroscope data. Extra two features are correlated with both the accelerometer and the gyroscope, so there are 561 features in total. The dataset consists of 7352 and 2947 training and test set samples, respectively. Furthermore, to study the power of different sensing units in activity recognition, we perform experiments with a single type of sensing unit (i.e., accelerometer or gyroscope) and two types of sensing units (i.e., accelerometer and gyroscope). Table 1 presents the dataset description, where “Acce & Gyro” refers to the use of both accelerometer and gyroscope.

### 4.2. Model Selection and Performance Metrics

As discussed above, the clustering-guided model takes various partitioning techniques and classification models as its building blocks. As for the data partition, we can do it manually or utilize a clustering algorithm. Since knowledge-driven methods have rather limited generalization to new and under-explored cases, we utilize clustering algorithms to perform the procedure in a data-driven way. Herein, we adopt the widely used *k*-means to partition data. We set the number of clusters returned by *k*-means to be the number of predefined activities. In addition, we evaluate the choice of other clustering algorithms in the next section. As for building the hierarchical activity recognizer, we can use the same classification model or different classification models. The two schemes are called *homogeneous* and *heterogeneous schemes*. In the study, we evaluate both cases. The naïve Bayes (NB), *k* nearest neighbor (KNN), support vector machine (SVM), and decision tree (DT) algorithms are adopted as the classification models. For KNN, we choose the nearest neighbor with the Euclidean distance metric. For SVM, the linear kernel is adopted. The four classification models are widely used in many existing studies [6,16,38]. For performance metrics, *accuracy*, *precision*, *recall*, and *F*1 are used to assess the power of an activity recognizer. *F*1, the harmonic mean of precision and recall.

## 5. Results and Analysis

### 5.1. Hierarchical Activity Recognizer

According to Algorithm 1, we first measure the confusion among activities and then build a clustering-guided hierarchical activity recognition model. Table 2 shows the activity confusion index between activities of UCI-HAR, where the column and row indicate the true activity and confusing labels of the activities. Furthermore, a confusion threshold *θ* is used to understand the confusion between similar activities. For example, if *θ* equals 0.03, the confusing activities of *go upstairs* include *walking* and *go downstairs*. If we set *θ* to be 0.005, the confusing activities of *go upstairs* include *walking*, *go downstairs*, *sitting*, and *lying*. Obviously, this makes it easier us not only to quantify the relationships among activities but also to correct the predictions of the top-level classifier.

**Figure 2:**
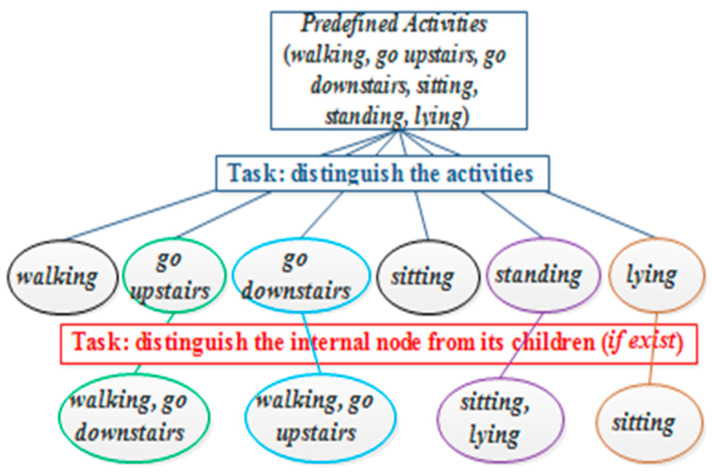
The clustering-guided hierarchical human activity recognizer on UCI-HAR.

Figure 2 presents the hierarchical human activity recognizer of UCI-HAR, where we empirically set the value of *θ* to be 0.01. As shown in Figure 2, the root node is to distinguish the six activities, the task of the internal node “*going upstairs*” is to distinguish the *walking*, *go upstairs*, and *go downstairs*, and the internal node “*standing*” aims to classify *standing*, *sitting*, and *lying*. Similarly, we can get the hierarchical activity recognizers for Gyroscope and Accelerometer datasets. After building the framework, the distinct classification models are integrated to train the top-level and second-level classifiers for optimizing a hierarchical activity recognizer.

### 5.2. Recognition Performance

**Figure 3:**
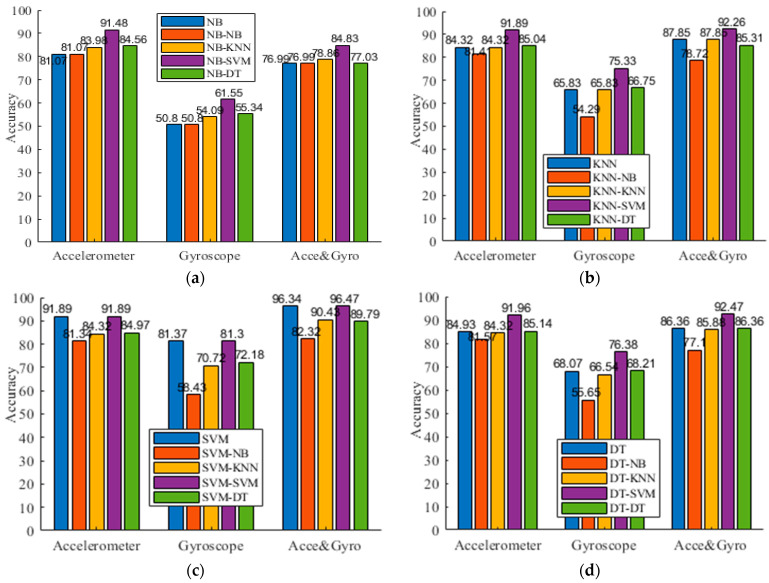
Accuracy comparisons on the three datasets. (**a**) NB; (**b**) KNN; (**c**) SVM; (**d**) DT.

We present experimental results of both the flat model and the hierarchical model, as shown in Table 3, Table 4 and Table 5 for three datasets, respectively. For the symbol “*P*-*Q*” of “the classifier” row, it means we use the classifier *P* at the top level and use the classifier *Q* at the second level. For example, NB-KNN indicates that we use NB at the top level, followed by KNN at the second level. To facilitate comparison, Figure 3 presents the comparison results of the four different activity recognition models. As shown in Figure 3, the usage of the accelerometer achieves better performance compared to the usage of the gyroscope. In addition, using both “Acce & Gyro” improves the accuracy. This indicates the priority of the Acce over Gyro in activity recognition and the complementary information shared by the gyroscope and accelerometer data. This is also consistent with previous studies. Exceptionally, we also observed that fusing gyroscope and accelerometer data did not guarantee the improved performance for all the cases, particularly for the use of NB. This is mainly because the redundant information provided by the 3-axis gyroscope and accelerometer destroys the conditional independence assumption. One possible way for further optimization is to conduct feature selection with filter, wrapper, or hybrid methods for filtering out irrelevant and redundant features. Second, we observe that the flat activity recognizers seldom classify a sample from dynamic activity into static activity (or vice versa). However, sometimes the wrong predictions are made in each group, which degraded the discriminant ability of an activity recognizer. This also motivates us to develop a hierarchical framework to recognize activities in a multi-step way. Third, except for the case of using NB at the second level, we observe that the proposed activity recognizer gets enhanced performance in many cases. This occurs because a hierarchical activity recognizer captures the confusion between types of activities and benefits from the finer step to classify confusing activities, which indicates the power of the clustering-guided model in discriminating activities that have similar sensor signals. Particularly, we have done this in a data-driven way instead of relying on expert knowledge to organize the activities of interest in a hierarchical structure. For example, NB-SVM obtains the 91.61% F1, compared with the 81.49% F1 of NB, 81.49% F1 of NB-NB, 83.95% F1 of NB-KNN, and 84.44% F1 of NB-DT in Table 3. On the UCI-HAR dataset, the best accuracy of the flat model is 96.47% achieved by SVM, and SVM-SVM improves it to 96.53%. This may indicate that SVM can better discriminate similar classes in finding an optimal separating hyperplane.

**Figure 4:**
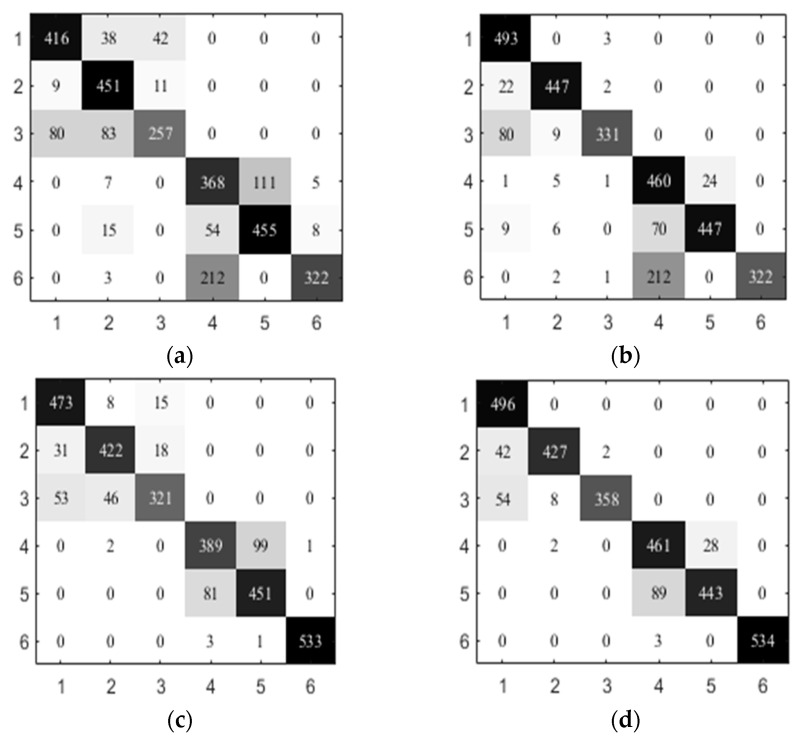

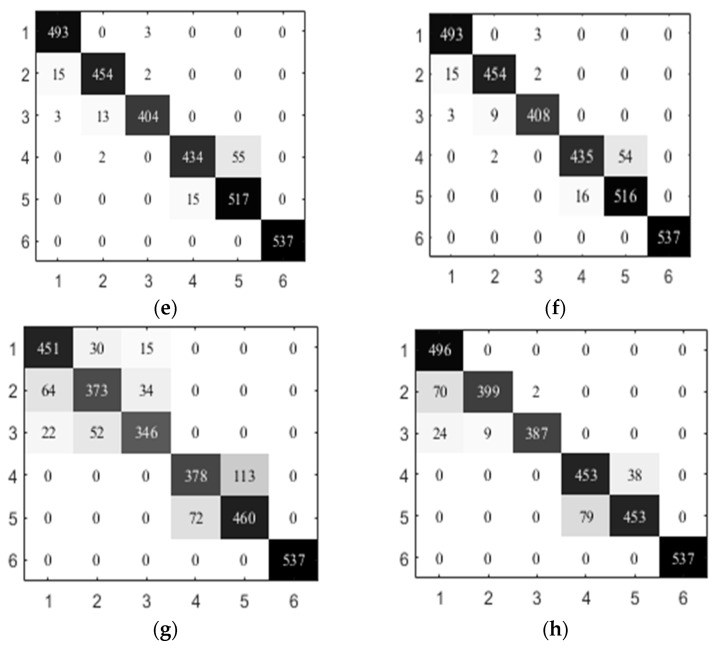
Comparison of the confusion matrix on UCI-HAR. (**a**) NB; (**b**) NB-SVM; (**c**) KNN; (**d**) KNN-SVM; (**e**) SVM; (**f**) SVM-SVM; (**g**) DT; (**h**) DT-SVM.

In addition, we present the confusion matrix of the activity recognizers to investigate the performance improvement. Due to the better performance of SVM, we give the results of NB, KNN, SVM, and DT and the comparative results obtained by using SVM at the second level. Figure 4 shows the results on UCI-HAR. The columns indicate the predicted labels and rows represent the actual labels. The used key-value pairs are: {1: *walking*, 2: *go-upstairs*, 3: *go-downstairs*, 4: *sitting*, 5: *standing*, 6: *lying*}. From Figure 4, we observe that the proposed hierarchical activity recognizer generally outperforms the flat model. For example, NB misclassifies 83 *go-downstairs* test samples into *go-upstairs*, and NB-SVM only makes 9 errors. Compared with SVM, SVM-SVM better discriminates between *go-downstairs* and *go-upstairs*. This indicates the superiority of a hierarchical activity recognizer in handling the activities that have similar sensor readings.

### 5.3. Evaluation of Different Confusion Thresholds

**Figure 5:**
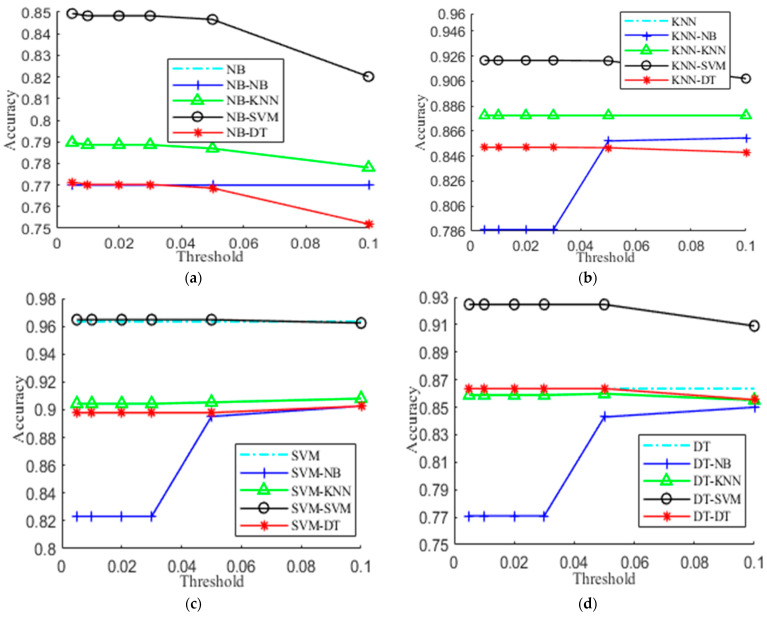
Accuracy vs. different thresholds. (**a**) NB; (**b**) KNN; (**c**) SVM; (**d**) DT.

The confusion threshold *θ* is a crucial parameter of the hierarchical activity recognizer, which decides the confusing activities of an activity. In this subsection, we experimentally evaluate the effect of varying *θ* on the performance. Based on our previous work, the candidates of *θ* include 0.005, 0.01, 0.02, 0.03, 0.05, 0.1, and 0.5. Figure 5 presents the experimental results on UCI-HAR. The X-axis gives the candidate values of *θ* and the Y-axis shows its accuracy. For Figure 5a, the curves of NB and NB-NB exactly coincide, and so is the case with KNN and KNN-KNN in Figure 5b. From Figure 5, we see that the performance of the hierarchical activity recognizer is closely related to *θ* and 1% is a reasonable choice that achieves comparable or higher accuracy in most cases. Particularly, a small value of *θ* tends to get a densely connected graph, and a larger value of *θ* could lead to sparse connections between the activities at the second level, which decreases the hierarchical model to a flat model. Hence, the accuracy tends to generally decrease with the increase of *θ* expect the case of KNN-NB. Actually, we could choose the value of *θ* in a broad range such as between 0.01 and 0.05. This greatly relieves us from parameter tuning. It should be noticed that SVM achieves better performance at the second level.

### 5.4. Evaluation of Different Number of Clusters

**Figure 6:**
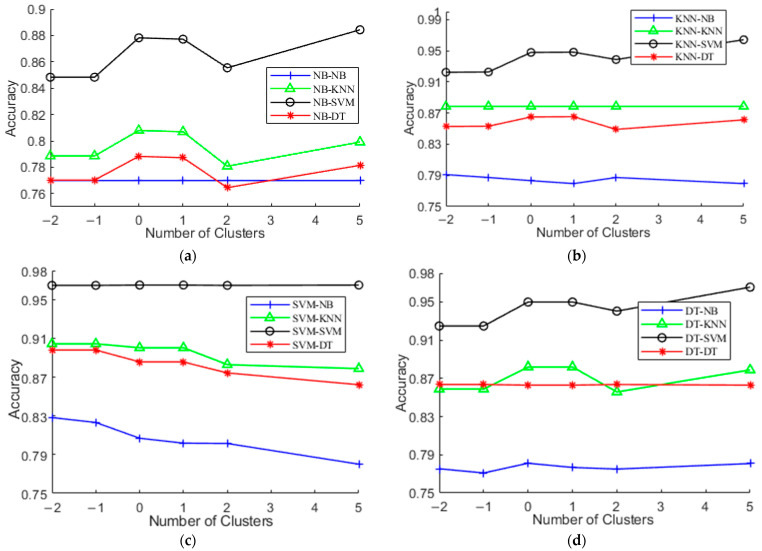
Recognition accuracy vs. different number of clusters. (**a**) NB; (**b**) KNN; (**c**) SVM; (**d**) DT.

In this subsection, the impact of the different number of clusters on the performance of hierarchical activity recognition models is studied. Figure 6 shows the experimental results on UCI-HAR. The X-axis refers to the number of clusters returned by *k*-means and the Y-axis is the accuracy obtained. Particularly, the values of the X-axis denote the values relative to the number |*L*| of predefined activities. For example, −1 means that the number of clusters of *k*-means equals |*L*| − 1, 0 indicates that the number of clusters equals |*L*|, and 1 means that the number of clusters equals |*L*| + 1. From Figure 6, we observe that setting the number of clusters to be the number of predefined activities generally obtains satisfactory accuracy, although there is no consistent trend between accuracy and |*L*|. This is most probable because this value corresponds to the true distribution of the dataset. Using the value helps relieve users from selecting the hyperparameter values for model optimization.

### 5.5. Evaluation of Different Distance Metrics

**Figure 7:**
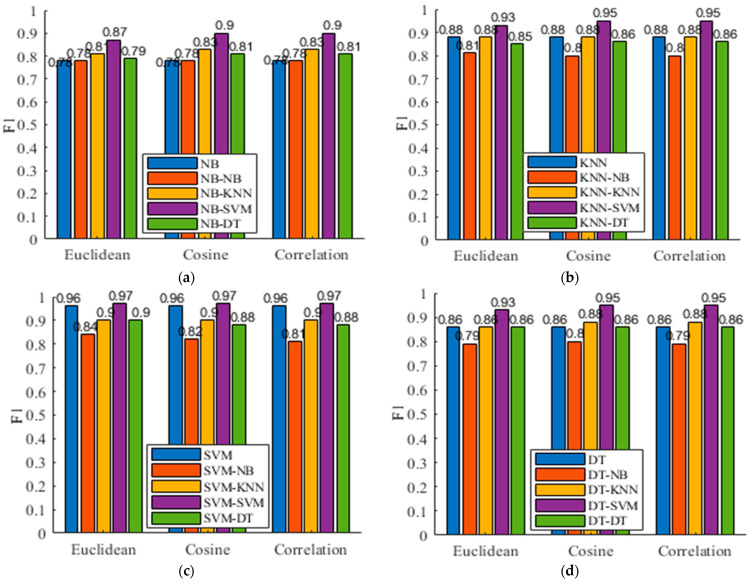
F1 vs. different distance metrics of k-means. (**a**) NB; (**b**) KNN; (**c**) SVM; (**d**) DT.

The distance metric is an important factor that determines the results of a clustering algorithm. We herein explore another two metrics (i.e., cosine and correlation) to calculate the distance between samples and further compare their performance with that of the Euclidean metric. Table 6 presents the experimental results, where the bold text indicates the best results. In addition, Figure 7 gives the corresponding F1. We observe that the use of the three metrics obtains comparable results. For example, in terms of SVM-SVM, the Euclidean, cosine, and correlation metrics obtain the 96.47%, 96.51%, and 96.51% accuracy, respectively. For DT-DT, the results are 86.36%, 86.29%, and 86.29% accuracy for the three metrics. This shows its robustness to the choice of distance metrics. We also observe that the use of the three metrics gets improved accuracy compared with the flat model. This demonstrates the effectiveness of the clustering-guided hierarchical model.

### 5.6. Evaluation of Different Clustering Algorithms

For the proposed framework, different clustering algorithms, besides *k*-means, can be integrated into it and function as its first component. In this subsection, we investigate another two commonly used clustering algorithms (i.e., *k*-medoids and agglomerative clustering), where the former chooses data points as the centers, and the latter builds a hierarchy of clusters. Specifically, according to the previous results, the number of clusters is set as the number of predefined activities. The Euclidean distance metric is used for *k*-means and *k*-medoids and applies the Ward’s method to calculate linkage of the agglomerative clustering.

**Figure 8:**
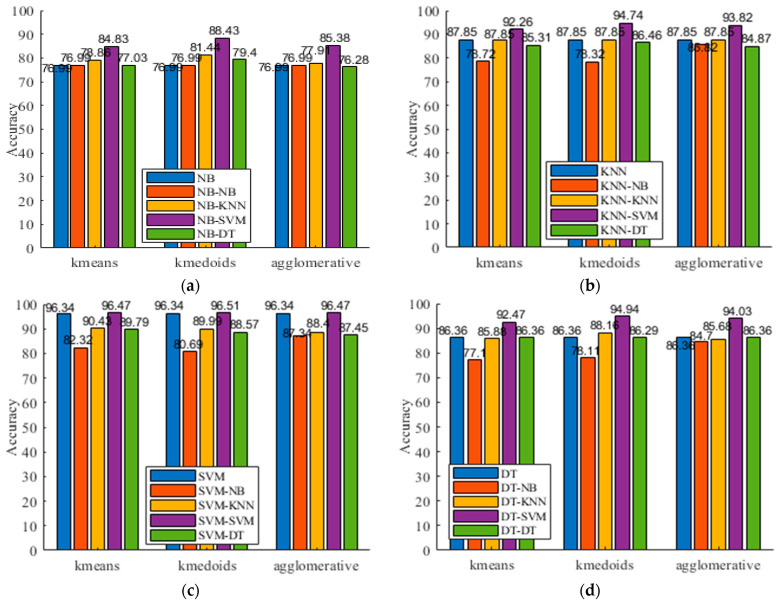
Recognition accuracy vs. different clustering algorithms on UCI-HAR. (**a**) NB; (**b**) KNN; (**c**) SVM; (**d**) DT.

Table 7 presents the corresponding results on UCI-HAR. In the table, the row “agglomerative” refers to the results when agglomerative clustering is used. The bold text indicates the best F1 of each group. In addition, Figure 8 gives the accuracy. From Table 7 and Figure 8, we observe that the hierarchical activity recognizer leads to enhanced recognition rates whichever clustering algorithm is used. We also observe that the use of *k*-means and agglomerative clustering obtains comparable performance and that the use of *k*-medoids slightly outperforms its competitors. For example, when NB is used as the top-level classifier, the best F1 of *k*-medoids, agglomerative clustering, and *k*-means are 0.9029, 0.8666, and 0.8683, respectively. If SVM is chosen as the top-level classifier, the F1 (0.9657) of *k*-medoids is higher than the F1 (0.9653) of both *k*-means and agglomerative clustering. The possible reason is that *k*-medoids takes data points rather than the average of the points in a cluster as the centers. This is consistent with the defined cluster confusion index and activity confusion index, where we use the majority voting principle to determine the confusing data points.

## 6. Conclusions

Accurately automating the recognition of human activities plays a prominent role in various applications like elderly care, ambient intelligence, smart homes, and human computer interactions. Training an activity recognition model poses difficulties in data training, modeling, and evaluation. Particularly, in the inter-subject and intra-subject variations, there are activities of interest that have similar sensor signals, which confuses an activity recognizer and result in degraded accuracy. In this study, a data-driven hierarchical activity recognition framework is proposed to quantify the confusion among activities and to build a hierarchical activity recognition model for improving the overall accuracy. Finally, the comparative analysis on the datasets is conducted, and the results show the success ratio of the grouping of the predefined activities in a specific application and its superiority over its competitors. In addition, we experimentally study the role of the two components within the framework from the aspects of sensitivity analysis of the confusion threshold, the choice of clustering algorithms and distance metrics, and the number of used clusters, which indicates its effectiveness.

In instantiating the framework, we can incorporate different clustering algorithms as well as classification models into it, where there are two hyperparameters (i.e., the number of clusters and the confusion threshold) that require us to assign their values. Although experimental results have shown its robustness, it is expected to adaptively set their values based on the data characteristics. This involves further studies for a comprehensive analysis. Second, the hierarchical framework brings larger computation costs, so there is a tradeoff between the recognition accuracy and resource constraints in deploying the model in edge devices.

## Figures and Tables

**Table 1:** The UCI-HAR dataset description.

Sensing Units	The Number of Features		
	Time-Domain	Frequency-Domain	Total
Accelerometer	164	184	348
Gyroscope	106	105	211
Acce & Gyro	272	289	561

**Table 2:** Class confusion index on UCI-HAR.

	Walking	Upstairs	Downstairs	Sitting	Standing	Lying
Walking	0	629	597	0	0	0
Upstairs	0	822	251	0	0	0
Downstairs	0	137	849	0	0	0
Sitting	0	1	0	0	1236	49
Standing	0	0	0	0	1374	0
Lying	0	10	0	0	164	1233

**Table 3:** Recognition performance on the Accelerometer dataset.

**Model**	**NB**	**NB-NB**	**NB-KNN**	**NB-SVM**	**NB-DT**	**KNN**	**KNN-NB**	**KNN-KNN**	**KNN-SVM**	**KNN-DT**
Accuracy	81.07	81.07	83.98	**91.48**	84.56	84.32	81.41	84.32	**91.89**	85.04
Precision	80.38	80.38	83.72	**91.50**	84.29	84.03	80.70	84.03	**91.88**	84.73
Recall	82.63	82.63	84.19	**91.72**	84.58	84.55	82.85	84.55	**92.16**	85.08
F1	81.49	81.49	83.95	**91.61**	84.44	84.29	81.76	84.29	**92.02**	84.90
**Model**	**SVM**	**SVM-NB**	**SVM-KNN**	**SVM-SVM**	**SVM-DT**	**DT**	**DT-NB**	**DT-KNN**	**DT-SVM**	**DT-DT**
Accuracy	91.89	81.34	84.32	**91.89**	84.97	84.93	81.57	84.32	**91.96**	85.14
Precision	91.87	80.64	84.03	**91.88**	84.67	84.63	80.87	84.03	**91.95**	84.82
Recall	92.16	82.77	84.55	**92.16**	85.01	84.99	83.03	84.58	**92.24**	85.19
F1	92.02	81.69	84.29	**92.02**	84.84	84.81	81.94	84.30	**92.09**	85.01

**Table 4:** Recognition performance on the Gyroscope dataset.

**Model**	**NB**	**NB-NB**	**NB-KNN**	**NB-SVM**	**NB-DT**	**KNN**	**KNN-NB**	**KNN-KNN**	**KNN-SVM**	**KNN-DT**
Accuracy	50.80	50.80	54.09	**61.55**	55.34	65.83	54.29	65.83	**75.33**	66.75
Precision	51.92	51.92	55.04	**62.83**	56.34	66.08	55.18	66.08	**75.70**	66.91
Recall	55.00	55.00	61.76	**71.04**	62.32	66.41	60.67	66.41	**77.97**	69.50
F1	53.42	53.42	58.20	**66.69**	59.18	66.25	57.79	66.25	**76.82**	68.18
**Model**	**SVM**	**SVM-NB**	**SVM-KNN**	**SVM-SVM**	**SVM-DT**	**DT**	**DT-NB**	**DT-KNN**	**DT-SVM**	**DT-DT**
Accuracy	**81.37**	58.43	70.72	81.30	72.18	68.07	55.65	66.54	**76.38**	68.21
Precision	**81.73**	59.43	71.04	81.66	72.39	68.17	56.57	66.68	**76.61**	68.29
Recall	**81.64**	64.17	72.36	81.60	73.41	68.24	62.09	69.47	**78.60**	68.34
F1	**81.69**	61.71	71.70	81.63	72.90	68.20	59.20	68.05	**77.59**	68.32

**Table 5:** Recognition performance on the UCI-HAR dataset.

**Model**	**NB**	**NB-NB**	**NB-KNN**	**NB-SVM**	**NB-DT**	**KNN**	**KNN-NB**	**KNN-KNN**	**KNN-SVM**	**KNN-DT**
Accuracy	76.99	76.99	78.86	**84.83**	77.03	87.85	78.72	87.85	**92.26**	85.31
Precision	76.88	76.88	78.90	**85.13**	77.08	87.44	78.63	87.44	**92.08**	84.80
Recall	79.23	79.23	83.48	**88.60**	81.09	87.96	82.86	87.96	**93.06**	85.98
F1	78.04	78.04	81.12	**86.83**	79.03	87.70	80.69	87.7	**92.57**	85.39
**Model**	**SVM**	**SVM-NB**	**SVM-KNN**	**SVM-SVM**	**SVM-DT**	**DT**	**DT-NB**	**DT-KNN**	**DT-SVM**	**DT-DT**
Accuracy	96.34	82.32	90.43	**96.47**	89.79	86.36	77.10	85.88	**92.47**	86.36
Precision	96.26	82.16	90.01	**96.42**	89.40	85.99	76.95	85.40	**92.38**	85.99
Recall	96.52	85.58	90.91	**96.65**	89.80	86.31	80.78	86.85	**93.13**	86.31
F1	96.39	83.84	90.46	**96.53**	89.60	86.15	78.82	86.12	**92.75**	86.15

**Table 6:** Performance of different distance metrics for *k*-means on UCI-HAR.

**Model**	**NB-NB**		**NB-KNN**		**NB-SVM**		**NB-DT**		**KNN-NB**		**KNN-KNN**		**KNN-SVM**		**KNN-DT**	
Metrics	Acc	F1	Acc	F1	Acc	F1	Acc	F1	Acc	F1	Acc	F1	Acc	F1	Acc	F1
Euclidean	**76.99**	0.780	78.86	0.811	84.83	0.868	77.03	0.790	**78.72**	0.807	**87.85**	0.877	92.26	0.926	85.31	0.854
Cosine	**76.99**	0.780	**80.69**	0.829	**87.72**	0.897	**78.72**	0.807	78.32	0.802	**87.85**	0.877	94.77	0.950	86.50	0.864
Correlation	**76.99**	0.780	**80.69**	0.829	**87.72**	0.897	**78.72**	0.807	77.94	0.796	**87.85**	0.877	**94.81**	0.950	**86.53**	0.865
**Model**	**SVM-NB**		**SVM-KNN**		**SVM-SVM**		**SVM-DT**		**DT-NB**		**DT-KNN**		**DT-SVM**		**DT-DT**	
Metrics	Acc	F1	Acc	F1	Acc	F1	Acc	F1	Acc	F1	Acc	F1	Acc	F1	Acc	F1
Euclidean	**82.32**	0.838	**90.43**	0.905	96.47	0.965	**89.79**	0.896	77.10	0.788	85.88	0.861	92.47	0.928	**86.36**	0.862
Cosine	80.69	0.820	90.02	0.899	**96.51**	0.966	88.57	0.883	**78.11**	0.798	**88.19**	0.882	**94.98**	0.951	86.29	0.861
Correlation	80.18	0.813	90.02	0.899	**96.51**	0.966	88.57	0.883	77.67	0.792	**88.19**	0.882	**94.98**	0.951	86.29	0.861

**Table 7:** F1 of different clustering algorithms on UCI-HAR.

**Model**	**NB**	**NB-NB**	**NB-KNN**	**NB-SVM**	**NB-DT**	**KNN**	**KNN-NB**	**KNN-KNN**	**KNN-SVM**	**KNN-DT**
*k*-means	0.7803	0.7803	0.8112	0.8683	0.7903	0.877	0.8069	0.8770	0.9257	0.8538
*k*-medoids	0.7803	0.7803	0.8350	**0.9029**	0.8122	0.877	0.8019	0.8770	**0.9495**	0.8640
agglomerative	0.7803	0.7803	0.7941	0.8666	0.7763	0.877	0.857	0.8770	0.9398	0.8480
**Model**	**SVM**	**SVM-NB**	**SVM-KNN**	**SVM-SVM**	**SVM-DT**	**DT**	**DT-NB**	**DT-KNN**	**DT-SVM**	**DT-DT**
*k*-means	0.9639	0.8384	0.9045	0.9653	0.8960	0.8615	0.7882	0.8612	0.9275	0.8615
*k*-medoids	0.9639	0.8201	0.8986	**0.9657**	0.8829	0.8615	0.7978	0.8818	**0.9509**	0.8607
agglomerative	0.9639	0.8732	0.8838	0.9653	0.8731	0.8615	0.8453	0.8574	0.9423	0.8615

## Data Availability

Not applicable.
